# Role of NIHR HTA clinical trials on subsequent systematic reviews

**DOI:** 10.1186/1745-6215-16-S2-P171

**Published:** 2015-11-16

**Authors:** James Raftery, Amanda Young

**Affiliations:** 1 University of Southampton and University Hospital Southampton, Southampton, UK; 2 National Institute for Health Research (NIHR), Evaluation, Trials and Studies Coordinating Centre (NETSCC), University of Southampton, Southampton, UK

## Background

The Health Technology Assessment programme relies partly on systematic reviews for the identification of topics for the commissioning of clinical trials. However, the Programme does not examine the extent to which relevant systematic reviews, new or updates, report.

## Aims and Objectives

To review the HTA portfolio of published trials to determine the extent to which HTA funded trials are included in subsequent systematic reviews.

## Method

A cohort of published HTA trials to December 2011 (from the metadata HTA project) were reviewed. The Cochrane Library was searched for reviews containing HTA funded trials using the technology and disease as search terms.

## Results

The preliminary findings from the Cochrane Library found 28 trials out of the eligible 121 trials (excluding four pilot and feasibility studies) were found in a subsequent Cochrane systematic review. The weight of the included HTA trials varied widely (from 100% to 9%). In eight reviews, the trial had a weight of 100% indicating how the trial provided the only evidence for that particular comparison. However, of those 28 trials, only half showed superiority. For the rest, the 95% confidence interval included a measure of no difference. The implications of this will be discussed.

## Conclusions

The HTA Programme completes one side of a loop from a systematic review to the need for a new trial but not vice versa. The impact of a new trial on the subsequent systematic review is not examined. Only by completing this side of the loop can it be established whether further research is required.

